# Association of birth and periodontal disease in Bolivia, Chile and Colombia

**DOI:** 10.7705/biomedica.7418

**Published:** 2024-08-29

**Authors:** Brenda Yuliana Herrera-Serna, Olga Patricia López-Soto, Diego León Rendón-Blandón, Estefanía Alfonso-Galeano, Laura Vanessa Salgado-Yepes, Tatiana Chacón

**Affiliations:** 1 Grupo de Investigación en Salud Oral, Universidad Autónoma de Manizales, Manizales, Colombia Universidad Autónoma de Manizales Grupo de Investigación en Salud Oral Universidad Autónoma de Manizales Manizales Colombia; 2 Facultad de Odontología, Universidad Santo Tomás, Bucaramanga, Colombia Universidad Santo Tomás Facultad de Odontología Universidad Santo Tomás Bucaramanga Colombia

**Keywords:** Delivery, obstetric, premature birth, preterm birth, obstetric labor, premature, pregnancy, oral health, parto obstétrico, nacimiento prematuro, trabajo de parto prematuro, embarazo, salud bucal

## Abstract

**Introduction.:**

Preterm birth is a major medical, social, and economic problem that causes a large proportion of neonatal mortality and morbidity, has a high impact on the healthcare system, and affects family quality of life. The weight of newborns with mothers with periodontal disease is significantly lower compared to mothers not affected by this oral disease. This adverse outcome is considered a global public health problem based on epidemiological data.

**Objective.:**

To determine the association between the prevalence of preterm births and periodontal disease in Bolivia, Chile, and Colombia from 2000 to 2020.

**Materials and methods.:**

This ecological study considered the population of women in Bolivia, Chile, and Colombia, according to the prevalence of preterm births and periodontal disease discriminated by age. The study covered the period between 2000 and 2020. The search strategy within the Institute for Health Metrics and Evaluation investigative tool included prevalence, age groups, causes of preterm births and periodontal disease, context and locations, women, and rates. Statistical analysis included a simple linear regression between preterm births and periodontal disease for each age group within each country.

**Results.:**

Preterm birth rates were higher in the 15-19 years age group (Bolivia: 697,563; Chile: 844,864; Colombia: 804,126). The periodontal disease prevalence increased with age, as we observed in the 45-49 years group (Bolivia: 22,077,854; Chile: 34,297,901, Colombia: 32,032.830). According to age groups, the linear regression was statistically significant (p < 0.001) in all age groups for the Bolivian population over 30 years for the Colombian, and only in the 15-19 years group for the Chilean women.

**Conclusion.:**

An association was found between preterm births and periodontal disease in all age groups in Bolivia, only in the group of 15 to 19 years in Chile, and 30 years and up in Colombia over the 20-year period.

## RESUMEN

**Introducción.:**

El parto prematuro es un problema médico, social y económico importante, causa gran mortalidad y morbilidad neonatal, tiene un impacto importante en el sistema de salud y afecta la calidad de vida de las familias. El peso de los recién nacidos de madres con enfermedad periodontal es significativamente menor en comparación con los de madres no afectadas por esta enfermedad bucal. Este resultado adverso se considera un problema de salud pública global según los datos epidemiológicos.

**Objetivo.:**

Determinar la asociación entre la prevalencia de parto prematuro y la enfermedad periodontal en Bolivia, Chile y Colombia entre el 2000 y el 2020.

**Materiales y métodos.:**

Este estudio ecológico consideró las poblaciones de mujeres de Bolivia, Chile y Colombia, y la prevalencia de partos prematuros y enfermedad periodontal, discriminadas por grupos de edad. El estudio abarcó el período entre el 2000 y el 2020. La estrategia de búsqueda con la herramienta de investigación del *Institute for Health Metrics and Evaluation* incluyó prevalencia, grupos de edad, años entre 2000 y 2020, causas de parto prematuro y enfermedad periodontal, contexto y ubicaciones, mujeres y tasas. El análisis estadístico incluyó una regresión lineal simple entre parto prematuro y enfermedad periodontal para cada grupo de edad dentro de cada país.

**Resultados.:**

Las tasas de partos prematuros fueron mayores en el grupo de 15 a 19 años (Bolivia: 697.563, Chile: 844.864, Colombia: 804.126). La prevalencia de la enfermedad periodontal aumentó con la edad, particularmente en el grupo de 45 a 49 años (Bolivia: 22'077.854, Chile: 34'297.901, Colombia: 32'032,830). Según los grupos de edad, la regresión lineal fue estadísticamente significativa (p < 0,001) para todos los grupos evaluados de la población boliviana, en los grupos mayores de 30 años para las colombianas y solo en el grupo de 15 a 19 años para las mujeres chilenas.

**Conclusión.:**

Se encontró asociación entre el parto prematuro y la enfermedad periodontal en todos los grupos de edad en Bolivia, solo en el grupo de 15 a 19 años en Chile, y de 30 años y más en Colombia en el período evaluado de 20 años.

Preterm birth is a major medical, social, and economic problem that causes a large proportion of neonatal mortality and morbidity. Preterm and low birth weight babies have a high impact on the healthcare system and have affected the quality of life of families. The risk of infant mortality is higher with increased prematurity, mostly when birth occurs before 34 weeks of gestation ^1^.

Epidemiological studies show an increase in preterm births and low birth weight worldwide. According to a systematic review of 2019 ^2^, the percentage of preterm births worldwide is 10.6% (95% CI: 9-12). More than 20 million babies worldwide are born prematurely and with low weight. These data vary between countries and regions; less developed countries have higher rates of both conditions ^3^. Considering the high prevalence of preterm births, efforts to prevent or reduce its incidence appear to be inadequate, especially about risk factors and their control.

More than 20 years ago, Offenbacher *et al.*^4^ showed an association between periodontal pathogens and premature births. The researchers found that the weight of newborns of mothers with periodontal disease was significantly lower than the weight of babies with mothers without this oral pathology. This adverse outcome of pregnancy was associated with preterm births. Periodontal disease is considered a global public health problem based on the amount of epidemiological data found in different social and geographic groups ^5^ and the worldwide burden of non-communicable diseases ^6^. From the analyzed data, researchers estimated that 18.2% of cases of low birth weight and preterm births could be attributed to periodontal disease. They speculated that this pathology in pregnant women could be contributing to more cases of adverse obstetric outcomes than smoking or consuming alcohol. Other research has found similar associations ^7^. Although the etiology of these events in newborns is not precisely known, premature residual activation by inflammatory microorganisms or mediators is one of the likely mechanisms ^8^. In this regard, approximately 40% of pregnant women have periodontal disease ^9^. It is becoming clear that oral health status influences overall health and well-being. Thus, periodontal disease has been associated with several systemic alterations such as cardiovascular, respiratory, or endocrine conditions ^10^^-^^12^.

The impact of preterm birth and periodontal disease affects geographical contexts differently, and the literature review did not show much information for Latin America, so this gap needs to be filled to contextualize and update the data. This knowledge will contribute to the formulation of appropriate interventions initially from the individual scope of each pregnant woman to be treated until adapting them to a general population level. Because of the wide diversity offered by the Latin American region between and within countries, it was necessary to take some contexts with different characteristics as a reference. Some of the relevant aspects between countries such as Bolivia, Chile, and Colombia are life expectancy in years (74.2, 82.1, 82.7, respectively) ^13^, infant mortality rate in deaths per 1,000 live births (29.4, 6.9, 12.7, respectively) ^14^, and fertility rate (3.4, 1.7, 2.1, respectively) ^13^. Other metrics have a major presence in chronic, non-communicable pathologies and place the three countries in a type IIIa epidemiological profile of post-transition or early supra-existence, according to Omran *et al.*^15^.

Thus, this ecological study sought to determine the association between the prevalence of preterm birth and periodontal disease in Bolivia, Chile, and Colombia from 2000 to 2020.

## Materials and methods

### Design

An ecological study was developed to evaluate the prevalence of preterm births in multiple population groups in the presence of a related factor such as periodontal disease, making use of secondary sources available and freely accessible. This study is mixed because it combines time series and multiple group evaluations ^16^. The analytical time series allowed us to determine the changes in the two variables studied in the populations of women from Bolivia, Chile, and Colombia. The association between the prevalence of periodontal disease, as the main predictor, and those of preterm births was evaluated through multiple groups.

### Data collection

The time series choice from 2000 to 2020 was because the interest in knowing the health results of the policies implemented since 2000 by the World Health Organization (WHO) and the Pan-American Health Organization (PAHO) ^17^.

Since age is an important factor for both pathologies, the analysis by age was done separately considering the following groups: 15 to 19 years, 20 to 24, 25 to 29, 30 to 34, 35 to 39, 40 to 44, and 45 to 49. The group under 15 years was excluded since periodontal probing, resulting in comparable diagnoses, is not recommended for this population ^18^.

This study used data from the Global Burden of Disease Study conducted by the Institute for Health Metrics and Evaluation ^19^. The Global Burden of Disease Study collects, analyzes, systematizes, and estimates population health information on more than 300 diseases worldwide. It standardizes information comparable across countries based on sources and existing research. The Global Burden of Disease team of researchers and statisticians make robust estimates to provide the best currently available information for comparisons across populations and over time. The statistical estimation methods have been previously published ^20^^-^^22^.

Preterm delivery is defined as births before 37 gestational weeks, and periodontal disease is described as a clinical attachment loss of more than 6 mm and gingival pocket depth of more than 5 mm ^19^.

The information on the standardized prevalence rates per 100.000 habitants of preterm birth and periodontal disease in women according to age groups was extracted from the Global Health Data Exchange tool ^19^. The scale used (per 100.000 habitants) from the original database was respected to preserve comparability between the two variables and other pathologies, such as the purpose of the Global Burden of Disease study ^23^. The Global Burden of Disease estimates the incidence, prevalence, and mortality of 369 diseases and injuries for both sexes and 204 countries and territories.

The input data was drawn from censuses, health services, household surveys, disease notifications, vital records and statistics, disease registries, satellite images, and other sources ^23^. This study complies with the Statement of Guidelines for the Reporting of Accurate and Transparent Health Estimates (GATHER) ^24^ recommended by the WHO. Only secondary data from the public domain were used. Therefore, the study did not require ethical evaluation. However, the research ethics committee of the University where investigators work approved it (Act 023-109 November 18, 2020).

### Procedures

The search strategy for the Global Health Data Exchange tool included:

1. measure: prevalence;
2. age: according to the groups described;
3. year: each year between 2000 and 2020;
4. cause: preterm birth and periodontal disease;
5. location: Bolivia, Chile, and Colombia;
6. sex: female;
7. metric: rate.

The link for the search was: http://ghdx.healthdata.org/gbd-results-tool?params=gbd-api-2019permanent_link/a788d2d27a02178fb41b07991602f877.

### Analysis

Descriptive statistics were used to establish the prevalence of preterm births and periodontal disease in each age group during the selected period for each country. The trend prevalence was calculated using Prais Winsten's generalized linear regression model, which offers a correction for the serial autocorrelation often identified in population data measures ^25^.

The Prais Winsten is a linear regression model in which the variables are time-dependent, the errors are correlated and follow a first-order autoregressive process. In the univariate case, the model is Yt = ß0 + ß1xt + ut. Where the error term ut follows an autoregressive process: ut = put - 1 + et. The errors e" are independent and identically distributed: N (o, σ^2^) ^26^.

The logarithmic transformation of the dependent variables and the prevalence coefficients of preterm birth and periodontal disease were given to reduce the heterogeneity of the residual variance and were maintained during the analysis.

The independent variable in each case was the year of the historical series. Therefore, the trend estimation based on the coefficient b" of each applied regression equation was significant. To measure the annual percentage change (APC) of the prevalences, the equation used was APC = [-1 + 10b1] * 100, where b1 corresponded to the slope of the line obtained in the regression equation.

To calculate the confidence intervals: ΔIC = -1 + 10 (b ± t * SD). Where b is the annual growth rate. The values of b and the standard deviation (SD) were extracted from the regression analysis, and the value of t was provided by the t test. At the 5% significance level, the trend was considered stable when the regression coefficient did not differ from zero (p ≥ 0.05), increasing when the regression coefficient was positive and significant, and decreasing when the regression coefficient was negative and significant ^25^.

Simple linear regression analysis was performed between preterm birth and periodontal disease for each age group in each country. All analyses were carried out in the Stata™, version 17.

## Results

The results showed the prevalence rates per 100,000 inhabitants in each age group studied from 2000 to 2020. For the complete time series, preterm birth rates were higher in the younger age groups (15-19 years), while the prevalence of periodontal disease increased with age (table 1).

**Table 1:** Prevalence of preterm birth and periodontal disease in Bolivia, Chile and Colombia

Country	Variable	Mean	Standard deviation	CI 95%	
Bolivia	Preterm birth				
	15-19	697,563	19,153	657,475	- 737,652
	20-24	686,317	19,113	646,313	- 726,322
	25-29	681,927	19,098	641,955	- 721,901
	30-34	675,258	19,117	635,245	- 715,271
	35-39	666,441	18,966	626,745	- 706,138
	40-44	653,217	18,749	613,974	- 692,460
	45-49	635,632	18,437	613,974	- 692,460
	Periodontal disease				
	15-19	779,984	26,208	725,130	- 834,839
	20-24	2,555,805	136,913	2,269,241	- 2,842,372
	25-29	5,707,047	379,113	4,913,552	- 6,500,541
	30-34	9,780,492	633,641	8,454,267	- 11,106,721
	35-39	14,108,152	804,409	12,424,521	- 15,791,832
	40-44	18,415,803	863,576	16,608,312	- 20,223,285
	45-49	22,077,854	764,102	20,478,562	- 23,677,134
Chile	Preterm birth				
	15-19	844,864	10,213	823,486	- 866,242
	20-24	837,193	10,088	816,079	- 858,308
	25-29	833,817	9,991	812,905	- 854,730
	30-34	829,008	9,918	808,248	- 849,768
	35-39	822,380	9,761	801,948	- 842,812
	40-44	811,959	9,621	791,822	- 832,095
	45-49	795,958	9,431	776,220	- 815,696
	Periodontal disease				
	15-19	1,088,214	10,692	1,065,835	- 1,110,592
	20-24	3,981,056	36,188	3,905,313	- 4,056,825
	25-29	9,859,294	108,544	9,632,108	- 10,086,484
	30-34	17,637,243	229,355	17,157,235	- 18,117,292
	35-39	24,742,314	295,921	24,122,945	- 25,361,687
	40-44	30,359,002	293,595	29,744,523	- 30,973,575
	45-49	34,297,901	289,059	33,692,892	- 34,902,914
Colombia	Preterm birth				
	15-19	804,126	8,134	787,077	- 821,123
	20-24	790,565	7,907	774,017	- 807,113
	25-29	784,979	7,778	768,701	- 801,257
	30-34	777,764	7,741	761,566	- 793,962
	35-39	769,089	7,578	753,228	- 784,948
	40-44	756,603	7,395	741,125	- 772,080
	45-49	738,951	7,055	724,185	- 753,716
	Periodontal disease				
	15-19	1,320,269	54,469	1,206,265	- 1,434,273
	20-24	4,439,538	221,615	3,975,694	- 4,903,382
	25-29	9,820,872	456,147	8,866,146 -	10,775,635
	30-34	16,608,742	551,241	15,454,941	- 17,762,462
	35-39	23,159,991	528,571	22,053,682	- 24,266,367
	40-44	28,552,076	473,711	27,560,584	- 29,543,551
	45-49	32,032,832	401,158	31,193,262	- 32,872,478

An increase in preterm birth rates was evidenced between 2000 and 2020 in each age group. Preterm birth rates were higher in the 15 to 19 age group than in the rest of the groups. Chile and Colombia showed similar behavior in preterm birth rates in the same age group. Although the preterm birth rates of the older age group ^45^^-^^49^ decreased, Chile had a rate per 100,000 inhabitants higher than Bolivia and Colombia in this specific group. By the year 2020, the results were similar in the three countries. Although preterm births slightly decreased between the age groups, a growing trend was observed within each group. This finding was evident with the highest increase in the annual percentage change in the younger age group (15 to 19 years).

For periodontal disease, the highest rates were in the oldest age group (45 to 49 years) and were consistent between 2000 and 2020. In the three countries, from the 15 to 19 years group to the 30 to 34 age group, rates nearly doubled from one group to the other. Chile presented an increase of 27.05% from the first age group (15 to 19 years) to the second age group (20 to 24 years); from the second to the third group (25 to 29 years) the increase was 41.52%, and from the third to the fourth (30 to 34 years) the increase was 57.55%. In Colombia, from the first to the second group, the increase was 27.60%; from the second to the third group was 46.24%; and from the third to the fourth, 65.89%. In Bolivia from the first to the second group, the increase was 25.87%; from the second to the third group, 40.53%, and from the third to the fourth, 58.49%. In the case of Colombia, the increase among these groups was higher than in Chile and Bolivia (table 2).

In preterm births, an increasing trend was observed with an APC < 3.0 in the three countries. The periodontal disease trend was also increasing (APC up to 4.5). However, among the age groups, the older age had a rising APC in periodontal disease (table 2).

**Table 2:** Prevalence rate trend of preterm birth and periodontal disease per 100,000 habitants in Bolivia, Chile and Colombia according to age groups between

Preterm birth									
Country	Age group	Rate 2000 (CI 95%)	Rate 2020 (CI 95%)	APC 2000-2020 (CI 95%)	Trend	Rate 2000 (IC 95%)	Rate 2020 (IC 95%)	APC 2000-2020 (CI 95%)	Trend
Bolivia	15-19	368.58 (293.44 - 469.14)	461.42 (370.80 - 558.31)	2.615 (2.548 - 2.682)	Increasing	1,076.54 (481.51 - 1,943.93)	1,120.75 (503.91 - 2,017.10)	3.041 (3.029 - 3.050)	Increasing
	20-24	358.91 (283.69 - 458.92)	450.46 (359.92 - 547.97)	2.604 (2.054 - 2.672)	Increasing	4,159.60 (2035.78 - 6,953.80)	4,344.52 (2,154.74 - 7,139.10)	3.629 (3.619 - 3.638)	Increasing
	25-29	354.99 (279.73 - 454.53)	446.23 (355.81 - 543.06)	2.599 (2.531 - 2.668)	Increasing	10,262.20 (5,346.29 - 16,919.45)	10,665.42 (5,591.32 - 16,950.32)	4.019 (4.013 - 4.027)	Increasing
	30-34	348.90 (274.59 - 448.25)	439.23 (349.44 - 528.43)	2.592 (2.531 - 2.662)	Increasing	17,542.33 (9,796.76 - 26,983.11)	18,043.67 (10,180.97 - 27,568.87)	4.250 (4.244 - 4.256)	Increasing
	35-39	342.97 (268.83 - 441.82)	432.43 (343.52 - 528.43)	2.585 (2.515 - 2.655)	Increasing	24,078.32 (13,800.05 - 34,599.66)	24,548.27 (14,401.66 - 34,872.05)	4.467 (4.462 - 4.472)	Increasing
	40-44	334.56 (260.88 - 433.99)	421.81 (333.69 - 518.28)	2.575 (2.504 - 2.645)	Increasing	29,099.18 (17,187.90 - 39,056.87)	29,538.37 (17,843.65 - 39,500.87)	4.468 (4.462 - 4.472)	Increasing
	45-49	323.84 (249.45 - 421.14)	407.54 (319.92 - 503.20)	2,556 (2.489 - 2.630)	Increasing	31,305.48 (20,181.04 - 40,706.31)	31,821.09 (20,709.93 - 41,216.00)	4.450 (4.493 - 4.504)	Increasing
Chile	15-19	649.85 (546.71 - 762.72)	830.35 (677.68 - 935.55)	2.871 (2.833 - 2.909)	Increasing	895.49 (414.92 - 1,612.83)	967.00 (457.86 - 1,728.56)	2.970 (2.956 - 2.985)	Increasing
	20-24	643.65 (541.47 - 756.01)	823.32 (671.09 - 929.17)	2.867 (2.829 - 2.904)	Increasing	3,308.56 (1,617.33 - 5,530.10)	3,595.98 (1,806.17 - 6,162.45)	3.539 (3.519 - 3.560)	Increasing
	25-29	641.14 (538.72 - 753.52)	820.48 (668.17 - 926.30)	2.865 (2.828 - 2.903)	Increasing	7,967.57 (3,951.20 - 132,229.41)	8,715.74 (14,055.95 - 4,449.61)	3.919 (3.881 - 3.958)	Increasing
	30-34	636.81 (534.85 - 749.02)	815.70 (663.58 - 921.53)	2.862 (2.824 - 2.900)	Increasing	636.81 (534.85 - 749.02)	14,985.54 (7,793.87 - 23,799.25)	4.154 (4.099 - 4.209)	Increasing
	35-39	631.69 (530.15 - 745.27)	810.35 (658.27 - 916.11)	2.859 (2.821 - 2.898)	Increasing	19,867.07 (10,848.40 - 30,547.20)	21,155.65 (11,487.90 - 31,362.25)	4.308 (4.244 - 4.371)	Increasing
	40-44	623.12 (522.17 - 737.05)	800.97 (649.32 - 907.14)	2.853 (2.815 - 2.892)	Increasing	25,493.83 (14,301.29 - 36,584.54)	26,923.245 (14,753.46 - 37,681.70)	4.413 (4.357 - 4.471)	Increasing
	45-49	610.10 (508.29 - 722.50)	787.88 (635.62 - 894.27)	2.845 (2.807 - 2.885)	Increasing	29,487.85 (17,776.46 - 40,251.22)	30,761.33 (18,296.12 - 41,447.34)	4.476 (4.432 - 4.520)	Increasing
Colombia	15-19	580.62 (484.11 - 692.11)	725.95 (591.24 - 837.05)	2.814 (2.771 - 2.857)	Increasing	1,958.85 (976.85 - 3,513.98)	2,291.85 (1,001.39 - 3,550.77)	3.334 (3.254 - 3.414)	Increasing
	20-24	568.65 (471.52 - 679.05)	716.85 (582.27 - 827.14)	2.807 (2.762 - 2.852)	Increasing	7,092.96 (3,617.28 - 11,519.81)	7,886.42 (3,695.24 - 12,030.39)	3.879 (3.831 - 3.925)	Increasing
	25-29	564.09 (467.58 - 674.44)	713.41 (578.70 - 823.77)	2.803 (2.758 - 2.850)	Increasing	15,337.32 (8,183.08 - 22,974.57)	16,340.52 (8,566.50 - 22,994.38)	4.201 (4.180 - 4.224)	Increasing
	30-34	556.97 (460.73 - 667.59)	707.40 (572.59 - 816.73)	2.799 (2.752 - 2.846)	Increasing	23,274.33 (14,273.49 - 33,089.13)	24,284.70 (14,814.94 - 33,059.83)	4.377 (4.365 - 4.389)	Increasing
	35-39	550.14 (453.90 - 660.14)	701.44 (565.68 - 810.46)	2.795 (2.747 - 2.843)	Increasing	29,513.97 (18,346.16 - 39,458.65)	30,500.61 (19,088.99 - 40,026.17)	4.477 (4.470 - 4.485)	Increasing
	40-44	539.76 (444.77 - 648.57)	692.45 (556.93 - 802.77)	2.788 (2.739 - 2.837)	Increasing	34,206.31 (21,476.40 - 43,879.15)	35,159.94 (21,927.23 - 44,384.38)	4.540 (4.532 - 4.548)	Increasing
	45-49	525.92 (430.82 - 634.02)	680.68 (545.54 - 790.40)	2.779 (2.727 - 2.830)	Increasing	36,701.60 (24,594.03 - 45,660.72)	37,644.96 (25,585.08 - 46,868.72)	4.567 (4.562 - 4.578)	Increasing

APC: annual percentage change

* p = 0.000

The linear regression analysis between preterm births and periodontal disease showed an association in all age groups in Bolivia, reaching the highest association in the 15 to 19 years with r^2^ = 0.957, all with statistical significance (p = 0.000). The positive coefficients implied that, for each increased unit in periodontal disease, we expected an increase of 2,065 in the prevalence of preterm births in this group. Although the r^2^ values were decreasing in the other age groups -meaning a lower association between the two variables- it was undeniable that even in the 45 to 49 group, the strength of the association was high (r^2^ = 0.888).

In Chile, only the group of 15-to-19-year-olds showed a statistically significant association with an r^2^ = 0.651. In this age group, for each increased unit in periodontal disease, an increment of 1,760 was expected in the prevalence of preterm births, close to the values in Bolivia. Although the other age groups showed a positive association, this one was low, and the absence of statistical significance is consistent with the wide confidence intervals.

**Figure 1:**
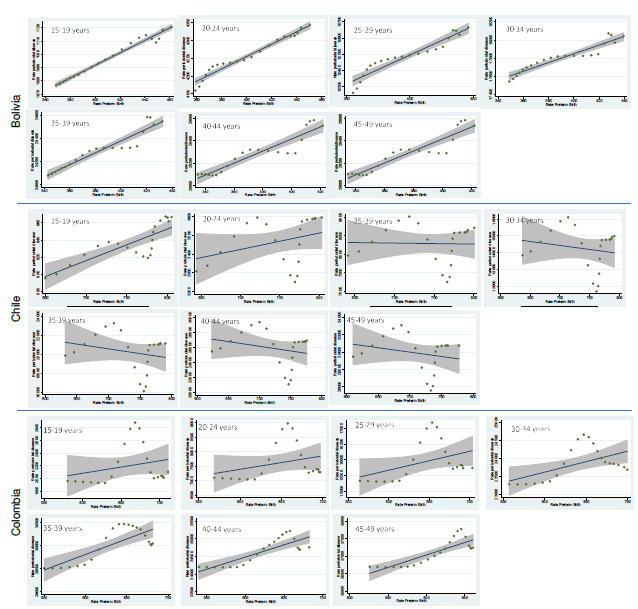
Association of preterm birth and periodontal disease in Bolivia, Chile and Colombia

In the case of Colombia, the association was observed with a r^2^ ranging from 0.305 to 0.707 in the 45 to 49 group. No association was found in the 15 to 19 group. Although, the 25 to 29 years old group had a p = 0.063 with an r^2^ = 0.170. On the other hand, for each increase in the prevalence of periodontal disease in the 45 to 49 group, a growth of 0.1 in the preterm birth prevalence was expected. Table 3 shows the results of the linear regression analyses. Figure 1 presents the linear regression graphs between preterm births and periodontal disease in each age group and country. The slopes reflect the positive association, and the confidence intervals are consistent with the absence of statistical significance in some groups.

**Table 3:** Association between preterm birth and periodontal disease according to countries and age groups

Country	Age group	r^2^	β (CI 95%)	p
Bolivia	15-19	0.977	2.065 (1.925 - 2.219)	0.000
	20-24	0.959	0.552 (0.498 - 0.606)	0.000
	25-29	0.905	0.255 (0.216 - 0.295)	0.000
	30-34	0.897	0.186 (0.155 - 0.216)	0.000
	35-39	0.908	0.177 (0.150 - 0.204)	0.000
	40-44	0.870	0.162 (0.132 - 0.191)	0.000
	45-49	0.888	0.135 (0.111 - 0.158)	0.000
Chile	15-19	0.651	1.760 (1.141 - 2.378)	0.000
	20-24	0.113	0.129 (-0.044 - 0.303)	0.136
	25-29	0.001	-0.002 (-0.046 - 0.042)	0.914
	30-34	0.032	-0.007 (-0.025 - 0.011)	0.436
	35-39	0.059	-0.006 (-0.016 - 0.006)	0.307
	40-44	0.062	-0.004 (-0.014 - 0.004)	0.276
	45-49	0.048	-0.005 (-0.016 - 0.006)	0.340
Colombia	15-19	0.082	0.038 (-0.229 - 0.097)	0.209
	20-24	0.089	0.019 (-0.010 - 0.488)	0.188
	25-29	0.171	0.028 (-0.002 - 0.582)	0.063
	30-34	0.305	0.052 (0.014 - 0.090)	0.009
	35-39	0.596	0.088 (0.053 - 0.123)	0.000
	40-44	0.673	0.097 (0.065 - 0.130)	0.000

## Discussion

Preterm births with their consequences, as well as periodontal disease, are important public health problems. The findings of this study showed an inverse relationship between age and preterm births and a direct relationship between age and periodontal disease. We found an association between preterm births and periodontal disease at all ages in Bolivia, only in the 15 to 19 group in Chile and from the age of 30 in Colombia (p < 0.001).

This ecological study provided population information of 20 years to expand data on a topic with conflicting conclusions from primary studies, such as the relationship between preterm birth and other adverse pregnancy outcomes and periodontal disease ^27^.

Regarding this relationship, it has been established that pregnancy accompanies significant and complex changes for both the mother and her developing baby. These changes increase a woman's susceptibility to various infections-including periodontal disease-thus, several studies have attempted to demonstrate the connection between oral microbiome and adverse pregnancy outcomes ^28^. Sex hormones can cause a significant change in the composition of the oral microbiome, leading to dysbiosis and an altered immune response ^29^^,^^30^. Chronic periodontal infections can provoke local and systemic inflammatory responses ^31^.

In the pathophysiological cycle of preterm births, low birth weight, preeclampsia and other pregnancy complications, activation of maternal inflammatory cell responses, cytokine release, and dysbiosis in the oral microbiota may play an explanatory role ^32^^,^^33^. It is important to consider that the variability in the data from the reviewed studies may be due to several factors such as dietary patterns, ethnicity, geographic location, and the research methodology used.

The highest prevalence of preterm births was observed in the 15 to 19 age group. This finding agrees with Njim *et al.*^34^ and with the concern to achieve Sustainable Development Goal 3: "Guarantee a healthy life and promote well-being at all ages in the Latin American region" ^35^. It has been considered that adolescent girls can be single, unemployed, and still in the education process, at best. Due to these financial and educational limitations, adolescents may not have adequate access to optimal prenatal care and have nutritional deficiencies, as well as risk factors such as smoking and alcohol consumption that could lead to increased preterm birth rates ^36^. However, the inverse relationship between age and the prevalence of preterm births is highlighted, implying an advance in control measures by related policies and healthcare teams.

A direct relationship between age and periodontal disease was evidenced. It coincides with the global data ^37^, and it is undeniable that oral health has been overlooked in policies aimed at combating chronic diseases ^38^. Oral diseases disproportionately affect the poorest and most marginalized groups in society and are strongly linked to socioeconomic status and broader social and business determinants ^5^. As the world intensifies efforts to control non-communicable diseases in the next decade, oral health can no longer be left behind and requires urgent and decisive actions ^38^.

The evaluated age groups showed an important change compared to some of the previously conducted studies, in which the age of the pregnant women was close to 25 ± 5 years ^39^^-^^42^. These studies have found a positive relationship between periodontal disease and preterm births. The chronic behavior of periodontal disease can serve as a platform for localized infection factors, immune response, inflammatory cascade, and systemic alterations and is expected to increase with age ^43^. In addition, Bolivia showed the highest net reproductive rate (1.6) of the three countries (Chile: 0.8, Colombia: 1.0) ^14^, favoring the probability of preterm births in a context of less education, lower income (Bolivia's human development index is the lowest in South America) ^44^ and lower access to first-level health services ^45^. Rural women with premature babies have also been found to have more severe periodontal disease ^40^. The comparison of the three countries included in this study showed a percentage of urbanization of 70.12 for Bolivia, 87.73 for Chile, and 81.43 for Colombia ^46^, with Bolivia being the country with the highest rurality.

It is well known that infant mortality, as a public health indicator, is more prevalent in rural areas ^47^, even in countries such as the United States ^48^. In turn, the lower levels of prenatal care coverage for indigenous women are widespread and cannot be explained solely by differences in wealth, education, or residence ^49^^,^^50^. Interventions at the community level, such as health education for pregnant women, show less inequality than those requiring access to services, such as delivery care. Regular monitoring of ethnic inequalities is essential to evaluate existing initiatives aimed at minority inclusion and to plan effective multisectorial policies and programs ^51^. These facts could explain the greater affectation evidenced over time in Bolivia.

This study showed some limitations. The first is that the annual percentage change analysis considers a community as the unit observed and analyzed, which could result in ecological fallacies if it is assumed causality or is interpreted individually. Furthermore, although many methods are used in the Global Burden of Disease study estimates to reduce bias -including corrections for misclassification, incompleteness, and redistribution of garbage codes- it can be difficult to completely avoid the uncertainty of data ^21^. The precision of the Global Burden of Disease estimates depends on the quality and quantity of the data sources, some of which depend on the mode of diagnosis, as in the case of periodontal disease. Therefore, the estimated prevalences for specific countries can lead to an overestimation or underestimation compared to the actual values. The results in the present study on the epidemiology of preterm birth and periodontal disease should be treated with care.

One of the strengths of this analysis is the use of data from the world's largest disease database. Despite the inherent limitations of the Global Burden of Disease study, large-scale epidemiological data continues to help health professionals related to maternal and child health and oral health, and key decision makers to guide research protocols, health policies and prioritization efforts to reduce the global burden of these major causes of morbidity.

As recommendations to practice, health professionals may consider strengthening multidisciplinary work. Preterm births are on the rise and periodontal disease among women seems to be out of control, so it is necessary to reinforce efforts among the treating personnel. Also, healthcare groups can take advantage of every moment of contact with pregnant women to generate a relevant memory. A pregnant mother who receives timely and quality care may be more likely to link her child to care services. This would generate adherence to health education. In the discussion forums for decision-makers, it is necessary to evaluate the effectiveness of current disease prevention programs and link impact evaluations, such as oral health programs, in the allocation of resources.

An association was found between preterm births and periodontal disease in all age groups in Bolivia, only in the 15 to 19 group in Chile and in those over 30 in Colombia. The prevalence of preterm births decreased between age groups during from 2000 to 2020, while there is a relationship between age and periodontal disease in Bolivia, Chile, and Colombia.

Although some age groups did not show a relationship between periodontal disease and preterm births, we can no longer ignore this important health topic during pregnancy or the years following childbearing. At this time, efforts could be directed toward learning how to encourage regular dental care before, during, and after childbirth. These findings suggest the need to strengthen first-level interdisciplinary health services and focus efforts on public policies to understand the differences between population groups to achieve a better rapprochement.
